# The Genetic Expression Difference of A2058 Cells Treated by Plasma Direct Exposure and Plasma-Treated Medium and the Appropriate Treatment Strategy

**DOI:** 10.3390/biomedicines13010184

**Published:** 2025-01-13

**Authors:** Chao-Yu Chen, Chung-Hsien Chou, Yun-Chien Cheng

**Affiliations:** 1 Department of Mechanical Engineering, National Yang Ming Chiao Tung University, Hsinchu 300, Taiwan; 2 Institute of Oral Biology, National Yang Ming Chiao Tung University, Taipei 112, Taiwan

**Keywords:** cold atmospheric plasma, reactive oxygen nitrogen species, cell viability, gene expression, cancer therapy

## Abstract

**Background/Objectives**: Cold atmospheric plasma (CAP) has been demonstrated as an adjustable device to generate various combinations of short-lived reactive oxygen and nitrogen species (RONS) and as a promising appliance for cancer therapy. This study investigated the effects of direct and indirect treatments of Argon-based CAP to cancer cells (A2058, A549, U2OS and BCC) and fibroblasts (NIH3T3 and L929) on cell viability. We also aimed to understand whether plasma-generated RONS were involved in this process using genetic evidence. **Methods**: The intensity of reactive species in the plasma gas and the concentrations of RONS in phosphate-buffered saline (PBS) and cell culture medium were measured. A viability assay was performed after the cells were treated by plasma in PBS and medium with various volumes to realize the lethal effects of plasma under different conditions. Diverse cells were treated in the same solution to compare the sensitivities of different cells to plasma treatments. The gene expression profiles of A2058 cells after the direct and indirect treatments were analyzed by next generation gene sequencing. Accordingly, we discovered the advantages of sequential treatments on cancer therapy. **Results**: The cumulative concentration of hydroxyterephthalic acid (HTA) revealed that the pre-existing OH radical (•OH) in PBS increased with the treatment durations. However, there was no significant increase in the concentration of HTA in culture medium. HTA was detected in the treatment interface of PBS but not medium, showing the penetration of •OH through PBS. The concentrations of H_2_O_2_ and NO_2_^−^ increased with the treatment durations, but that of NO_3_^−^ was low. The direct treatments caused stronger lethal effects on cancer cells under certain conditions. The fibroblasts showed higher tolerance to plasma treatments. From gene expression analysis, the initial observations showed that both treatments influenced transcription-related pathways and exhibited shared or unique cellular stress responses. The pre-treatments, especially of direct exposure, revealed better cancer inhibition. **Conclusions**: The anti-cancer efficiency of plasma could be enhanced by pre-treatments and by adjusting the liquid interfaces to avoid the rapid consumption of short-lived RONS in the medium. To achieve better therapeutic effects and selectivity, more evidence is necessary to find optional plasma treatments.

## 1. Introduction

Cold atmospheric plasma (CAP), a non-equilibrium ionized gas containing an abundant mixture of electrons, ions, neutral atoms and molecules including free radicals, excited species and metastable species, is a promising device for anti-cancer treatment in vitro and in vivo [1,2,3]. It has been demonstrated to have lethal effects on diverse kinds of cancer cells, including chemo-resistant cancer cells [4,5,6]. Plasma treatments trigger cancer cells to undergo apoptosis [7,8], inhibit cell migration [9], alter the interaction between cancer cells and the tumor microenvironment [10], and induce anti-tumor immune responses [11,12,13]. In addition to its effects on sterilization [14,15] and in promoting wound healing [16,17] and the potential applications combining plasma and other therapeutic methods such as chemotherapy [18,19], immunotherapy [20,21] and radiation [22], plasma can work as both an active and an adjunctive tool in cancer therapy.

When plasma is in operation, many reactive oxygen and nitrogen species (RONS), such as hydroxyl radicals (•OH), hydrogen peroxide (H_2_O_2_) and nitrogen oxides (NO_x_), are generated in the working gas or from the interaction between the plasma and the ambient air [23]. RONS can also be produced in liquids during plasma treatment [23]. The lifetimes of these RONS can be shorter than 1 s or longer than 1 h [24]. Since RONS are broadly involved in intercellular and intracellular signaling, the concentrations of RONS in cells and in their milieu affect cellular responses crucially [25]. Cancer cells contain more RONS than normal cells in order to take advantage of the function of RONS as secondary messengers to regulate cellular signaling for promoting proliferation and escaping apoptosis [25,26]. Therefore, the exogenous RONS provided by plasma can cause cancer cells to elicit apoptosis more readily than normal cells [27].

CAP treatment is usually performed with two main methods, direct and indirect treatment. With direct treatment, the target is exposed to plasma, and the physical factors of plasma, such as ultraviolet radiation and an electric field, plasma-generated short-lived reactive oxygen and nitrogen species (RONS) and long-lived RONS, have the opportunity to contact and influence cells. With indirect treatment, on the other hand, plasma-treated solution (PTS) is obtained from exposing bio-solutions to plasma to accumulate long-lived RONS and then is transferred to treatment targets. Researchers have found that not only direct irradiation but also PTS can be used to treat cancer cells by promoting cell apoptosis [28]. PTS has an advantage in medical application, as it can be produced in bulk amounts. Some PTSs can be stored in refrigerated or frozen condition for a time before usage [29], and thus this method provides a stabler and safer modality. Nevertheless, in PTS, only long-lived RONS still exist. Other active components in plasma might not be retained. Therefore, the understanding of the different effects of direct and indirect plasma treatments is important for the clinical development of further plasma treatments.

Dayun Yan et al. compared direct and indirect treatments of MDA-MB-231 cells and PA-TU-8988T cells cultured in Dulbecco’s modified Eagles medium (DMEM) with a helium (He)-based plasma jet and found that direct treatments exhibited a stronger effect on cell death [30]. Fariba Saadati et al. employed He plasma to treat B16-F10 melanoma cells cultured in DMEM supplemented with 15% (*v*/*v*) fetal bovine serum (FBS) in vitro and xenograft in vivo and confirmed that direct treatments caused potent cell death and decreased tumor size [31]. In contrast to the finding of Kyriakos Sklias et al. that, although indirect treatment of He/O_2_ plasma on CAL27 and FaDu carcinoma cells and normal cells (1Br3, NHK, and RPE-hTERT) in phosphate-buffered saline (PBS) showed selectivity, the direct treatment induced severe death in both cancer and normal cells [32], Inès Hamouda et al. found that direct treatment with He plasma notably reduced the cell viability of SaOS-2 sarcoma cells and MG63 osteosarcoma cells but not that of non-malignant osteoblast primary cells (hOBs) and bone marrow-derived mesenchymal stem cells (hMSCs) in 150 µL of AdvDMEM/10% FBS for 0.5 to 5 min. This selectivity was also observed under indirect treatments [33]. Sybille Hasse et al. treated 3D MNT-1 and SK-MEL-28 melanoma spheroids in RPMI1640/8% FBS with argon (Ar)-based plasma jet kINPen MED and discovered that significant declines in ATP levels were only observed in direct treatments [34]. The study of Mahdiyeh Bakhtiyari-Ramezani et al. reported that U-87 glioblastoma MG cells in vitro and glioblastoma multiforme in vivo were more sensitive to direct rather than to indirect treatment with He plasma but more sensitive to indirect rather than to direct treatment with Ar plasma [35]. In our previous study, it was also found that A2058 cells cultured in 3D hydrogel showed slight differences in viability when cells were directly exposed to plasma and treated with plasma-treated culture medium (PTM). The UV and electric fields generated in the system would not cause cell death, so the main influencing factors were likely the long-lived and short-lived RONS produced during the plasma exposure, and the slight differences might have been caused by short-lived RONS [36].

Some studies have investigated the utility and cellular effects of different PTSs prepared by culture medium and buffered solutions [37,38]. However, just a few studies describe the differences between direct and indirect treatments via different liquid layers [39]. Their effects with sequential treatments, for example, the direct or indirect treatment in addition to indirect treatment, were not compared. The whole picture is far from clear. In this study, we measured the cumulative concentration of HTA to evaluate the generation of •OH and measured the concentrations of H_2_O_2_, NO_2_^−^ and NO_3_^−^ in PTM and plasma-treated PBS (PTB). We studied the effects of direct and indirect treatments with an Ar-based CAP jet (Ar-CAP) on cell viability through medium or PBS. To further understand the cellular response to the two treatment methods, we analyzed high throughput gene expression profiles to interpret if their effects caused cellular responses to varying degrees or via different signaling pathways. A tactic for enhancing the effects of short-lived RONS on cancer therapy with sequential treatments was then determined. This study provides evidence of the effects of short-lived RONS on cancer therapy and provides a new direction in clinical treatment strategies.

## 2. Materials and Methods

### 2.1. Experimental Setup of Cold Atmospheric Plasma Device

**Figure 1:**
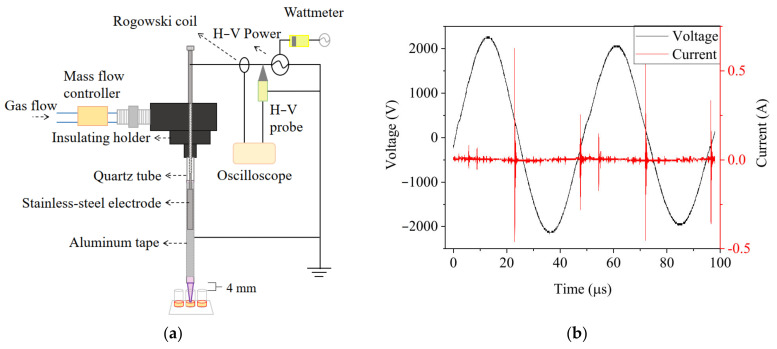
(**a**) The schematic diagram of the Ar-CAP system. (**b**) Voltage and current waveforms of Ar-CAP with 3-slm flow rate and 4.43-kVpp power supply.

The experimental setup of the argon-based CAP (Ar-CAP) device is shown in Figure 1. 

The plasma jet was fabricated with a quartz tube as the dielectric layer. Argon with a concentration of 99.99% was used as the working gas with a flow rate of 3 slm, set by a float flowmeter (0–5 slm, Yongxin Instruments, Hsinchu, Taiwan). Power with a discharge voltage of 4.43 kVpp at a frequency of 20.8 kHz was supplied to a slot-shaped stainless steel cylinder placed in the quartz tube as a high-voltage electrode. Aluminum foil was pasted outside the quartz as the ground electrode. The voltage and current were monitored and recorded with an oscilloscope (TDS1012B, Tektronix, Beaverton, OR, USA) through a high-voltage probe (P6015A, Tektronix, Beaverton, OR, USA) and Rogowski coil (IPC CM-100-MG, Ion Physics Corporation, Fremont, NH, USA). The emission spectra of excited species in plasma in the range of 180–900 nm were measured by optical emission spectrometry (OES, PI Acton SP 2500, Princeton Instruments, Trenton, NJ, USA). Imaging of the Ar-CAP was performed with a digital camera (Power Shot SX50 HS, Canon, Japan).

For treating solutions in 96-well plate for RONS measurements and cell culture, the distance between the jet outlet and the top of the well was 4.0 mm and the average distance between the outlet and liquid surface was 14.4, 13.7, 12.2 and 10.6 mm for 30, 50, 100 and 150 μL of solutions.

### 2.2. Measurement of 2-Hydroxyterephthalic Acid (HTA), Hydrogen Peroxide (H_2_O_2_), Nitrate (NO_2_^−^) and Nitrite (NO_3_^−^) Concentrations

HTA fluorescence assay was performed to measure the •OH generated during the plasma irradiations. The fluorescence intensity at 425 nm of HTA, formed from the interaction between terephthalic acid (TA, Sigma-Aldrich, St. Louis, MO, USA) and •OH in phosphate-buffered saline (PBS) or culture medium, was excited by 310 nm UV light and measured with an OES (Ocean Optic USB2000+, Ocean Optics, Dunedin, FL, USA). The absolute concentration of HTA was converted from the HTA calibration curve. The medium used in this study was DMEM/10% fetal bovine serum (FBS) without phenol red.

To observe the penetration of •OH in PBS and cell culture medium, TA-containing agarose gel was introduced to replace the plastic bottoms of 96-well plates. Plates were drilled to remove the plastic bottoms and the holes taped over. TA-containing agarose gel was prepared by mixing 1% (wt/vol) agarose (MB755-0100, GenedireX, Taoyuan, Taiwan) with 2 mM TA and then added to each 96-well plate as the plate bottom. PBS and DMEM/10% FBS medium in volumes of 30, 50, 100 and 150 μL were added for plasma treatments. After the treatments, the agarose gel was removed from the 96-well plates and the fluorescence intensity of the gel at 425 nm was measured by OES.

After the treatments with Ar-CAP on 30, 50, 100 and 150 μL of PBS or DMEM/10% FBS for 10, 20, 30, 40, 50 and 60 s, the concentration of hydrogen peroxide (H_2_O_2_) in the PBS or culture medium was measured using an Amplex Red hydrogen peroxide/peroxidase assay kit (Thermo Fisher Scientific, Waltham, MA, USA) and those of nitrite (NO_2_^−^) and nitrate (NO_3_^−^) were measured using a Cayman’s nitrate/nitrite colorimetric assay kit (Interchim, Montluçon, France) by following the standard procedures. The absorbances at 570 nm, 540 nm and 540 nm were detected with a microplate reader (Epoch, BioTek, Winooski, VT, USA) after the completion of the reactions for the measurements of hydrogen peroxide, nitrite and nitrate, respectively. The concentrations were quantified from their respective calibration curves.

### 2.3. Cell Culture

Human melanoma A2058 and BCC cells, lung adenocarcinoma A549 cells, osteosarcoma U2OS cells and mouse fibroblast NIH3T3 and L929 cells were cultured in DMEM supplemented with 10% fetal bovine serum (FBS, Gibco, New York, NY, USA), 100 U/mL penicillin and 100 mg/mL streptomycin (Corning, Corning, NY, USA) at 37 °C with 5% CO_2_.

### 2.4. Plasma Treatment of Cells

To understand the effects of the two methods of plasma treatments, namely, direct exposure (DE) and plasma-treated medium (PTM), and to make short-lived RONS function effectively in cancer inhibition, the methods were applied individually and as pre-treatments in sequential plasma treatments. Cells were treated in 4 groups: (1) DE, (2) PTM, (3) DE plus PTM and (4) PTM plus PTM.

Meanwhile, to understand the influences of applying with different treatment interfaces, PBS was used. However, due to the lack of essential nutrition for the cells, PBS was replaced with medium after 20 min. The procedures were as follows.

Cells were seeded into 96-well plates with a density of 8000 cells per well 24 h before plasma treatments for the viability assay. A density of 16,000 cells per well was seeded and grown to confluence for the mobility assay. For direct plasma exposure (DE), the medium was replaced with 30, 50, 100 or 150 μL of fresh medium or PBS and treated with Ar-CAP for 0, 10, 20, 30, 40, 50 and 60 s, denoted as DE0, 10, 20, 30, 40, 50 and 60. For indirect treatment, DMEM/10% FBS medium and PBS buffer with volumes of 30, 50, 100 or 150 μL were treated with Ar-CAP for 0, 10, 20, 30, 40, 50 and 60 s to prepare plasma-treated mediums 0, 10, 20, 30, 40, 50 and 60 (denoted as P0, 10, 20, 30, 40, 50 and 60) and plasma-treated buffers 0, 10, 20, 30, 40, 50 and 60 (denoted as PTB0, 10, 20, 30, 40, 50 and 60). PTM and PTB were left to stand at room temperature for 20 min and to replace the medium to treat the cells.

One of three conditions was implemented after plasma treatments: (1) cells were continuously incubated in treated medium, (2) cells were incubated in treated PBS for 20 min, and the PBS was replaced with fresh medium, or (3) cells were incubated in treated medium for 20 min and the treated solutions were replaced with P30. The cells were then left to culture for 18 h for scratch assay and 20 h for viability assay.

Cell viability was measured with a 3-(4,5-cimethylthiazol-2-yl)-2,5-diphenyl tetrazolium bromide (MTT, Thermo Fisher Scientific) assay. MTT solution at a concentration of 0.5 mg/mL was employed to replace the medium and to culture treated cells for 4 h in darkness. The formazan crystals were dissolved using dimethyl sulfoxide (DMSO, Sigma-Aldrich, St. Louis, MO, USA). The optical absorbance at 570 nm was measured with a microplate reader.

Cell migration was measured by scratch assay. A pipette tip was employed to create a scratch in confluent cells. After the treatment and incubation, treated cells were washed twice with PBS and fixed in 4% formaldehyde (Sigma-Aldrich, St. Louis, MO, USA) for 20 min. The fixed cells were stained with 0.5% crystal violet (Sigma-Aldrich, St. Louis, MO, USA) for 20 min and the images were acquired with a microscope (DM IL, Leica, Wetzlar, Germany) and a digital camera (DFC420 C, Leica, Wetzlar, Germany). The scratch area was defined as the basal area and the cell coverage rate of this area was analyzed quantitatively in the software ImageJ v.1.53t.

### 2.5. Gene Sequencing and Functional Analysis

Cells were seeded in 96-well plates with a density of 8000 cells per well 24 h before plasma treatments. Cells were treated in three groups. The first group was replaced with fresh medium without plasma treatment as the control. The second and third groups were treated with DE50 and P50, respectively. Cells were harvested after further culturing for 2.5 h. Total RNA was extracted with the RNeasy mini kit (Qiagen, Hilden, Germany) by following the standard procedures in the manual. The extraction quality was analyzed with a NanoDrop ND-1000 spectrophotometer (Thermo Scientific, Waltham, MA, USA) and an Agilent 2100 Bioanalyzer (Agilent Technologies, Palo Alto, CA, USA) for RNA integrity number (RIN). Total RNA with RIN > 8.0 and A260/A280 = 1.8–2.0 was used for purification with a strand mRNA Library Preparation Kit (Illumina, San Diego, CA, USA) and fragmentation. The cleaved RNA fragments were reverse transcribed to generate a cDNA library. Each enriched cDNA library was sequenced by NovaSeqX (Illumina Inc.) with 2 × 150 bp paired-end nucleotide reads.

Differential gene expression (DGE) analysis was performed using DESeq2 in the R statistical environment. The normalization of gene counts was performed using the median of ratios method followed by differential expression analysis. Genes with a Log2 fold change (Log2 FC) greater than 1 or less than −1 and *p* ≤ 0.05 were considered to be significantly differentially expressed. For functional analysis, significant genes were input into Ingenuity Pathway Analysis (IPA) to identify enriched canonical pathways and functions.

## 3. Results

### 3.1. Optical Emission of Ar-CAP

**Figure 2:**
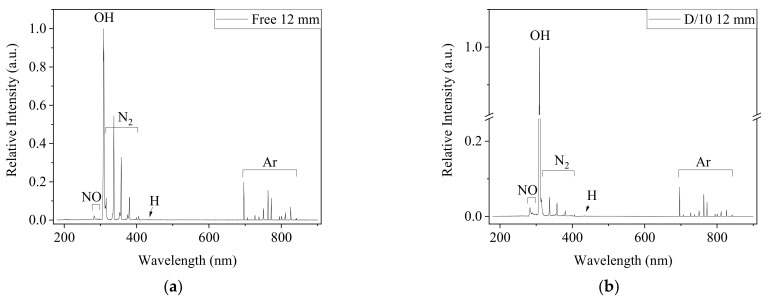
The OES spectra from 180 to 900 nm captured 12 mm from the outlet of Ar-CAP in the free jet (**a**) and on DMEM/10% FBS medium (**b**). The distance between the outlet and medium surface was 12 mm.

The excited species generated in Ar-CAP in air and in medium were monitored by OES. The major features were similar in the two spectra (Figure 2). In the UV region of 230–290 nm, the emission of NO formed joined peaks. The high intensities of the emission lines at 309 and 315 nm revealed the emission from hydroxyl radicals (•OH), and the peaks at 337, 357, 380 and 406 indicated the emission from nitrogen (N_2_). A weak signal from hydrogen at 434 nm was also detected. The measurements from 696 to 842 nm depicted Ar-active species. No signals from active oxygen at 777, 844 or 926 nm were observed.

Nevertheless, the relative emission intensities under two conditions showed different patterns. All emission intensities of Ar and NO increased, and the intensity at 309 nm was much stronger in the spectrum captured when CAP was the treating medium than that captured in ambient air without physical contact. However, the signals from N_2_ did not show significant changes. Overall, the plasma intensity was enhanced when the plume contacted the liquid.

**Figure 3:**
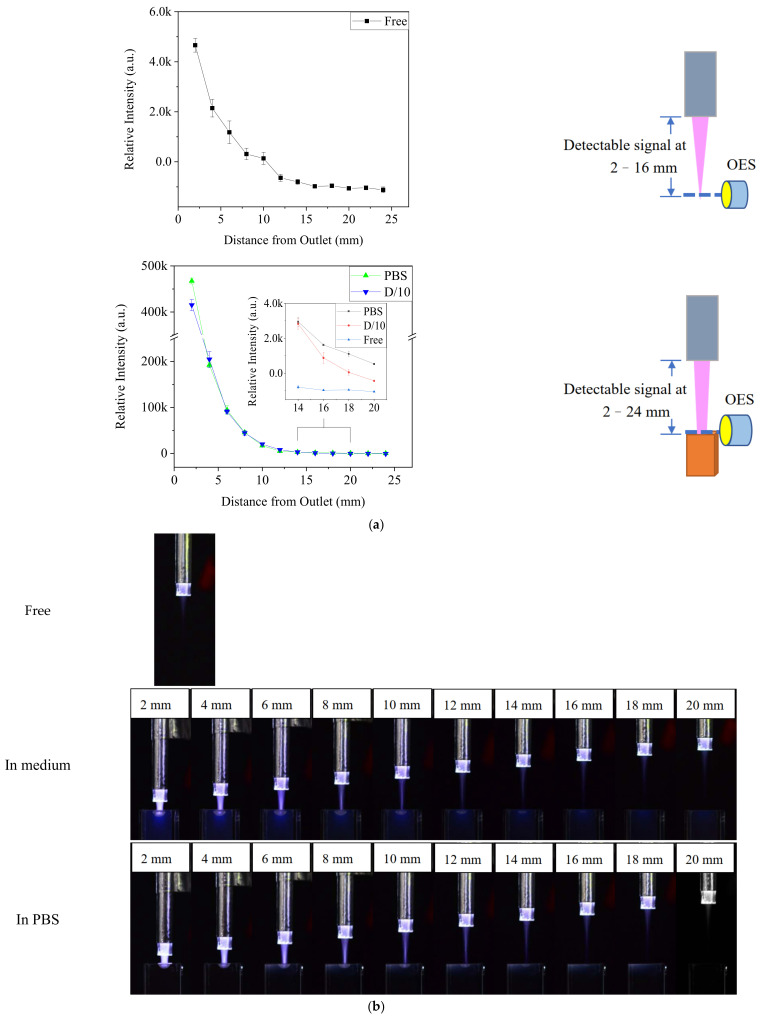
(**a**) The optical emission intensity at 309 nm in plasma with and without contacting solution at different distances. (**b**) Images of free Ar-CAP and Ar-CAP in medium and in PBS with different distances from the outlet to the liquid surface.

To determine whether the enhancement of plasma was affected by the distance of treatment, the •OH emissions at 309 nm were measured at various distances from the jet outlet, but with a smaller aperture to avoid overexposed signals at the outlet (Figure 3). When the distance between the outlet and medium surface was 2 mm, the •OH signal was almost two orders stronger than that in the free jet. Furthermore, the •OH signal could be detected at a distance of 24 mm in medium, whereas it was the same as the background value at 16 mm in free plasma. The enhanced plasma on the liquid surface was visible in the images of the Ar-CAP being performed in gas phase and on the liquid (Figure 3b).

### 3.2. HTA Measurement After the Treatments of Ar-CAP

**Figure 4:**
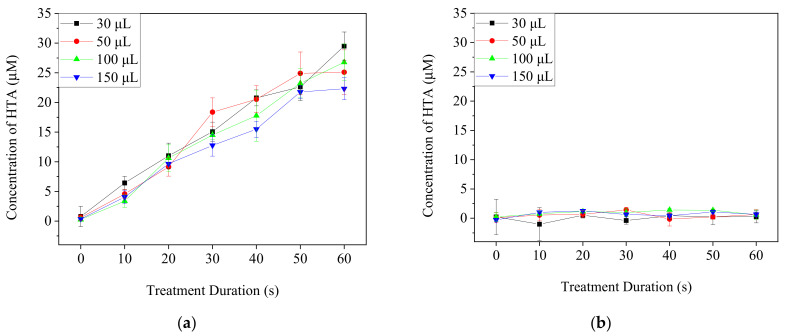
The concentrations of HTA in PBS (**a**) and DMEM/10% FBS medium (**b**) after the treatments with Ar-CAP for 0 to 60 s.

TA-containing PBS and DMEM/10% FBS medium with different volumes in 96-well culture plates were treated by Ar-CAP for 0–60 s. The fluorescence intensity of HTA at 425 nm in solution was measured and then calibrated to the concentrations (Figure 4). The concentration of HTA in PBS increased proportionally to the prolonged treatment duration of the plasma. However, no significant increases in the fluorescence of HTA were detected from plasma-treated medium in any of the conditions. Therefore, the HTA concentration was assumed to be very low.

**Figure 5:**
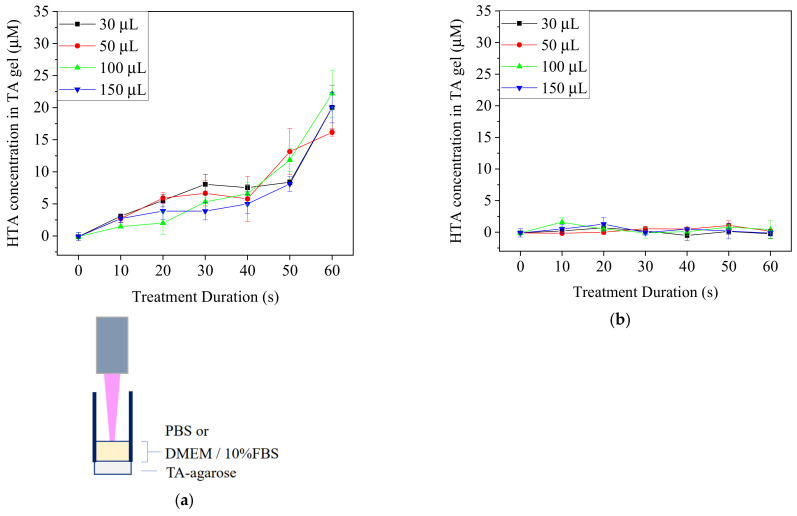
TA-containing agarose gel was used at the bottom of a 96-well plate. PBS and medium were treated with Ar-CAP for 0 to 60 s. The fluorescence intensities of HTA in agar beneath PBS (**a**) and DMEM/10% FBS medium (**b**) were measured at 425 nm after the treatments with Ar-CAP and calibrated to concentration.

To confirm if plasma-generated •OH could reach cells during the treatments in spite of the depletion, TA-containing agarose gel was used to replace the plastic bottoms of 96-well plates and then removed for the fluorescence detection of HTA (Figure 5). The increased fluorescence intensity at 425 nm of the agarose gel beneath PBS showed that •OH penetrated the PBS layer by 30 μM to 150 μM and contacted the agarose gel. In contrast, the intensity of agarose gel beneath the medium treated for 60 s did not differ significantly from that of the control of 0 s. These findings revealed that •OH might react with the components in the medium and hardly penetrate through the medium to reach the cell culture area in the plate.

### 3.3. Concentration of Long-Lived RONS in Ar-CAP-Treated PBS and Medium

**Figure 6:**
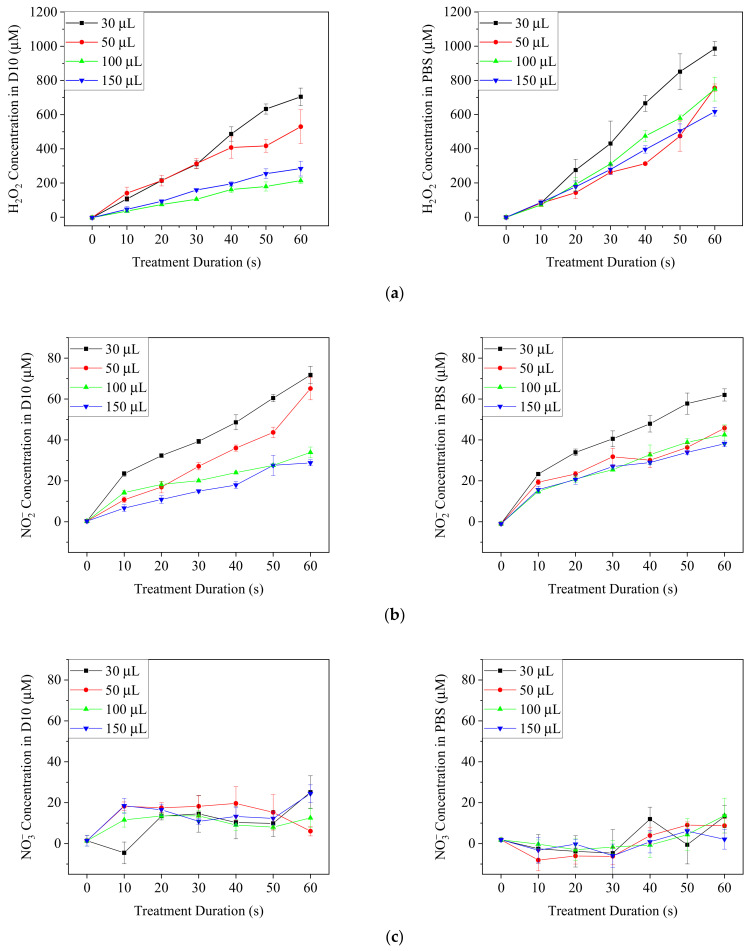
The concentrations of H_2_O_2_ (**a**) NO_2_^−^ (**b**) and NO_3_^−^ (**c**) in plasma-treated DMEM/10% FBS medium (D10) and PBS after plasma treatments for 0 to 60 s.

DMEM/10% FBS medium and PBS were exposed to Ar-CAP for 0 to 60 s, and the concentrations of H_2_O_2_ and NO_x_ were measured (Figure 6). The concentrations of H_2_O_2_ and NO_2_^−^ increased nearly linearly with elongated treatment times in both medium and PBS. By contrast, the production of H_2_O_2_ in PBS was higher than that in culture medium, but the concentrations of NO_2_^−^ in the two solutions were similar. The concentrations of NO_3_^−^ were low and did not increase significantly along with the treatment durations. The low concentration resulted in some negative values during the calculation from the colorimetric method. In acidic solutions, nitrous acid can convert into nitrate through a series of chemical reactions [40]. However, the buffer property of the medium maintains the solution pH value at around 7, which makes the reaction inefficient [40]. This might explain the low concentration of nitrate in PBS and medium in our system.

### 3.4. Viability of A2058 Cancer Cells with Direct and Indirect Ar-CAP Treatments

**Figure 7:**
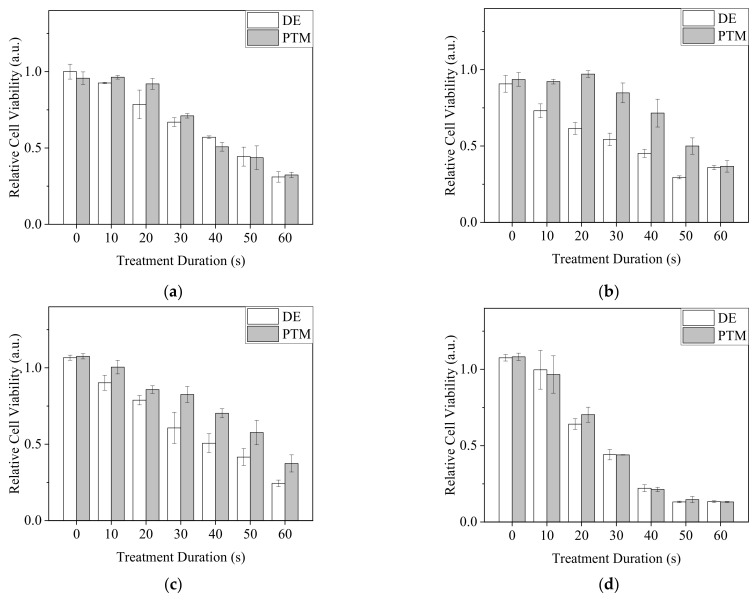
The viability of DE- or PTM-treated A2058 cells. Cells were treated by DE or PTM with medium volumes of (**a**) 30 μL, (**b**) 50 μL, (**c**) 100 μL and (**d**) 150 μL.

For direct exposure (DE) treatment, A2058 cells were exposed to Ar-CAP with medium volumes of 30, 50, 100 and 150 μL for 0 to 60 s as DE0 to DE60 and continuously cultured in treated medium for 20 h. For plasma-treated medium (PTM) treatment, 30, 50, 100 and 150 μL of medium were exposed to plasma for 0 to 60 s as P0 to P60 and then used to culture A2058 cells for 20 h. Cell viability was then determined by MTT assay (Figure 7).

In 30 and 150 μL of medium, as the DE and PTM treatment times were prolonged, the viability of A2058 cells decreased. However, the effects of DE and PTM did not show significant differences. In 50 and 100 μL of medium, DE showed stronger lethal effects than those of PTM. The treatments at the volume of 150 μL showed a severe lethal effect in comparison with those of other volumes, possibly because of the enhanced plasma intensity, which generated more RONS and the peroxidation of organic molecules in the medium, thus causing stronger oxidative stress on the cells.

**Figure 8:**
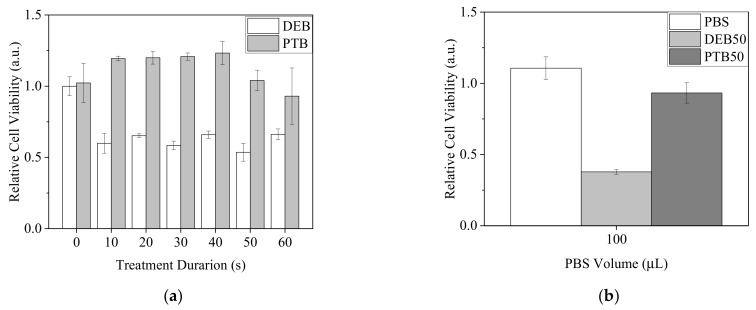
Viability of A2058 cells with treatments of direct plasma exposure in PBS and plasma-treated PBS. (**a**) Cells were treated by DEB and PTB with a PBS volume of 30 μL for 0 to 60 s. (**b**) Cells were treated by DEB50 and PTB50 with a PBS volume of 100 μL. Cell viability was measured after 20 h recovery in complete medium.

Since •OH was demonstrated to penetrate 30 μL of PBS in 96-well plates, we assumed it was possible that most of the plasma-generated short-lived RONS could reach the cells under this condition. To understand if plasma-generated short-lived RONS, including •OH, influenced the viability of A2058 cells, cells were treated with direct plasma exposure in PBS (DEB) for 0 to 60 s or 0 to 60 s plasma-treated PBS (PTB) with the PBS volume of 30 μL. After 20 min of incubation, treated PBS was replaced by complete medium. Cells were further cultured for 20 h. MTT assay was carried out to measure cell viability (Figure 8). Intriguingly, all DEB treatments significantly reduced cell viability, but the effects did not exhibit obvious changes regardless of the treatment durations. In contrast, the treatments of PTB had minor effects or increased cell viability (Figure 8a). Similarly, when the volume of 100 μL was used with plasma exposure for 50 s for DEB and PTB treatments, DEB showed a higher lethal effect on A2058 cells (Figure 8b). From the results, we assumed that short-lived RONS affected the cells strongly with DEB treatment.

### 3.5. Mobility of A2058 Cancer Cells with Direct and Indirect Treatments of Ar-CAP

**Figure 9:**
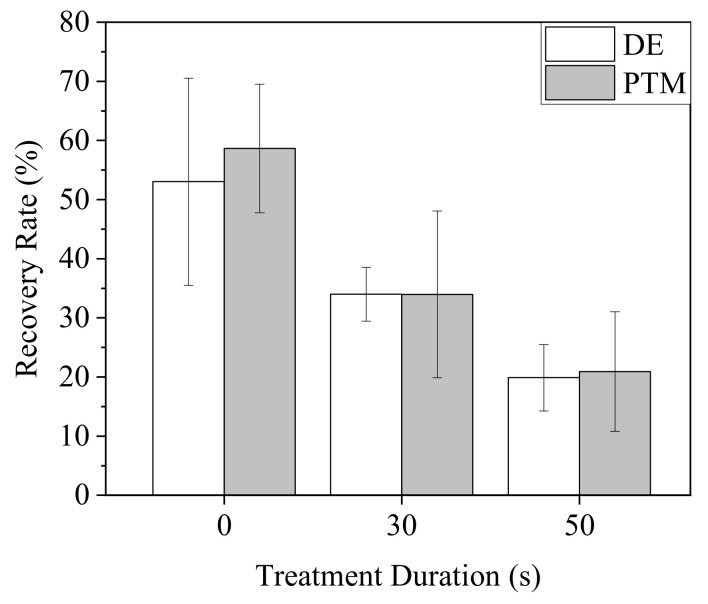
Mobility of A2058 cells after plasma treatments. Cells were treated by DE and PTM with the medium volume of 100 μL and continuously cultured for 18 h.

To clarify the effects of direct and indirect treatments of Ar-CAP on cell mobility, a scratch assay was performed on A2058 cells treated by DE and PTM (Figure 9). When A2058 cells were continuously cultured in treated medium for 18 h, the mobility of both DE- and PTM-treated cells decreased as the treatment durations increased, in similar trends.

### 3.6. Viability of A549, U2OS and BCC Cancer Cells with Direct and Indirect Treatments of Ar-CAP

To elucidate the effects of direct and indirect treatments on the viability of cancer cells, A549, Hep3B, U2OS and BCC cells were treated by DE and PTM.

**Figure 10:**
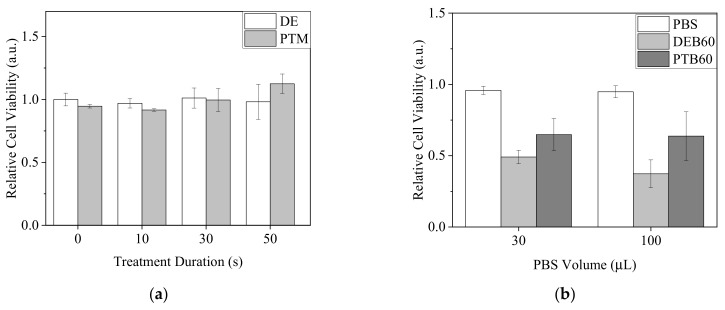
Viability of A549 cells after plasma treatments. (**a**) Cells were treated by DE and PTM with a medium volume of 100 μL and continuously cultured for 20 h. (**b**) Cells were treated by DEB60 and PTB60 with PBS volumes of 30 and 100 μL for 20 min. The medium was replaced with complete medium and the cells were further incubated for 20 h.

A549 revealed a tolerance to our Ar-CAP treatments in 100-μL DMEM/10% FBS medium. Neither DE nor PTM treatments had significant lethal effects (Figure 10a). However, when A549 cells were treated by DEB60 and PTB60 for 20 min and recovered in complete medium for 20 h, cell viability decreased after both DEB and PTB treatments. DEB showed stronger effects than PTB (Figure 10b).

**Figure 11:**
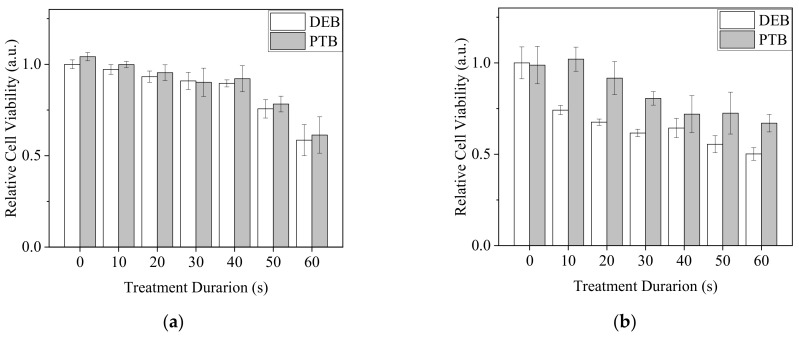
Viability of U2OS (**a**) and BCC (**b**) cells with the treatments of DEB and PTB in 30 μL of PBS for 20 min and recovered in medium for 20 h. The Ar-CAP exposure times were 0 to 60 s.

Since plasma-generated RONS might interact with the complex components in the medium before they contacted the cells, we treated U2OS and BCC cells with DEB and PTB in 30 μL of PBS (Figure 11). Nevertheless, plasma treatments in PBS exhibited different effects on the two types of cancer cells. With treatment times of 50 s or longer, plasma treatments decreased U2OS cell viability. DEB and PTB treatments resulted in similar effects (Figure 11a). In comparison, the viability of BCC cells revealed an inverse proportion to treatment time. DEB treatments led to a lower viability than the PTB treatments did (Figure 11b).

### 3.7. Viability of NIH3T3 and L929 Fibroblasts with Direct and Indirect Treatments of Ar-CAP

**Figure 12:**
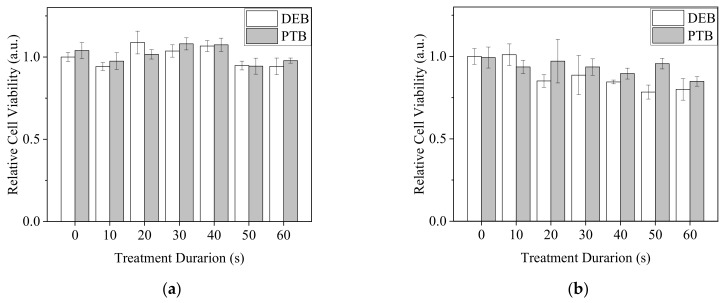
Viability of NIH3T3 (**a**) and L929 (**b**) fibroblasts with the treatments of DEB and PTB in 30 μL of PBS for 20 min and recovery in medium for 20 h. The Ar-CAP exposure times were 0 to 60 s.

To elucidate the influence of direct and indirect plasma treatments on normal cells, two fibroblasts, NIH3T3 and L929 cells, were treated with DEB and PTB (Figure 12). Neither DEB nor PTB brought on significant changes in the viability of NIH3T3 cells. Viability remained higher than 0.9, as compared with the untreated control at 1.0. In comparison, plasma treatments slightly decreased the viability of L929 cells. DEB treatments of more than 50 s reduced viability to lower than 0.85.

### 3.8. Gene Expression Analysis of Ar-CAP-Treated A2058 Cells

**Figure 13:**
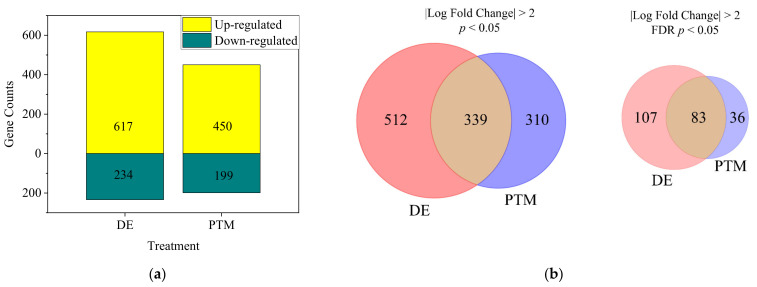

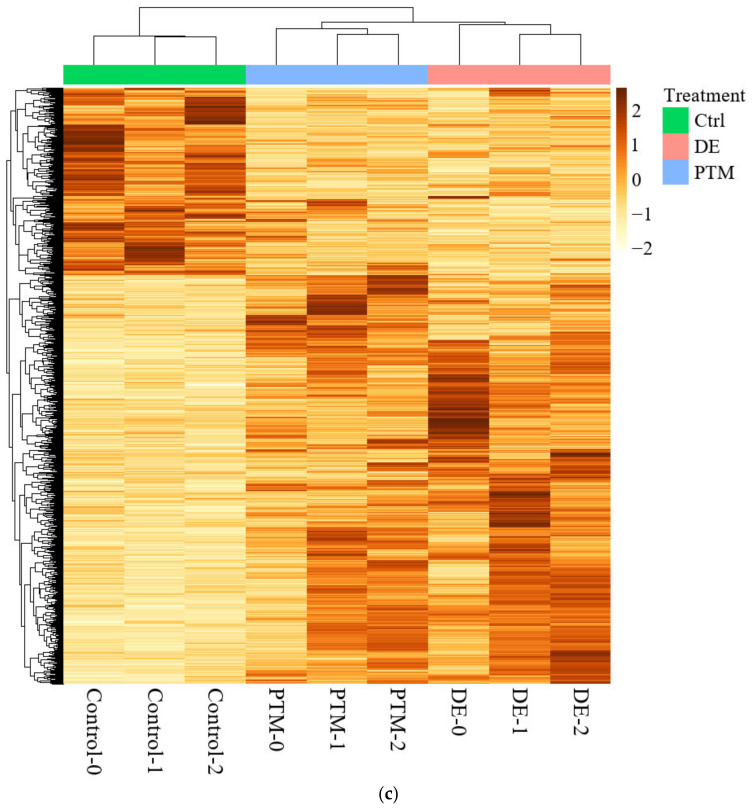
(**a**) The numbers of significant differentially expressed genes (DEGs) of DE- and PTM-treated A2058 cells with fold changes of more than 2 and *p* values of less than 0.05. (**b**) Venn diagrams showing the intersection of D- and PTM-treated DEGs, filtered by fold change of more than 2 and significance at *p* < 0.05 (**left**) or FDR-adjusted *q* < 0.05 (**right**). (**c**) The heatmap of significant DEGs. Deeper colors represent higher expression, and lighter colors, lower expression. Columns represent each condition, including control, PTM and DE.

Although the majority of •OH in the medium could potentially be eliminated, considering the complicated environment of the human body and open wounds in clinical application, A2058 cells were treated by Ar-CAP directly with 100 μL of DMEM/10% FBS for 50 s or with P50 and incubated for 2.5 h. Total RNA was sequenced to obtain expression profiles. Based on the analytic criteria of fold changes of ≥2 and *p* < 0.05, when cells were treated with DE, 617 genes were upregulated and 234 genes were downregulated. When cells were treated with PTM, 450 genes were upregulated and 199 genes were downregulated (Figure 13).

The DEG analysis, using *p*-value filtering (*p* < 0.05, fold change ≥ 2), identified 512 DE-specific genes, 310 PTM-specific genes and 339 shared between treatments. While FDR (q-value) filtering refined the results, *p*-value-based filtering captured a broader range of significant genes. DE-specific genes were more biologically interpretable, reflecting the combined effects of short- and long-lived RONS in DE treatment. In contrast, PTM-specific genes—driven by long-lived RONS—might represent delayed or subtler cellular stress responses before cell death.

### 3.9. Impact of Ar-CAP Treatment and Comparison Between DE and PTM

To further explore the biological effects of plasma treatments, we conducted an Ingenuity Pathway Analysis (IPA) pathway and function analysis, which revealed significant impacts on various cellular and physiological pathways and functions. The analysis highlighted both the overall impact of Ar-CAP treatment and the specific differences between the two treatment modalities.

**Figure 14:**
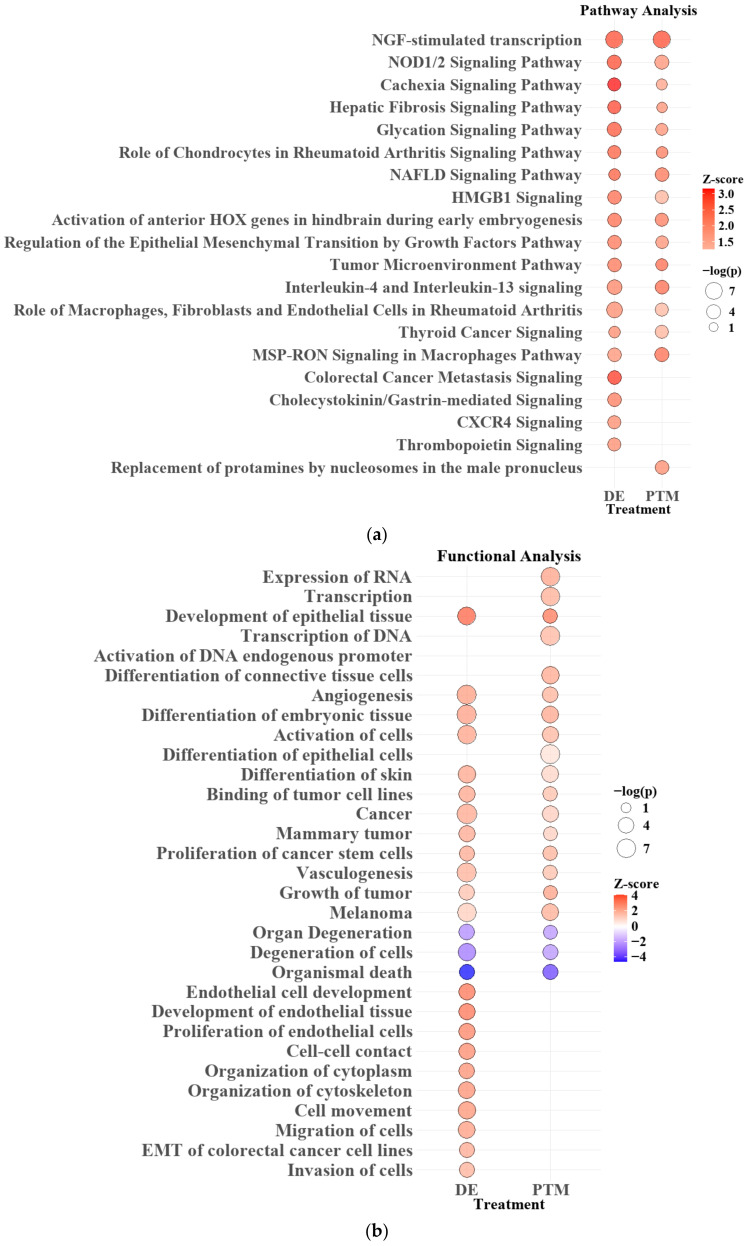
Pathway (**a**) and functional (**b**) analysis for DE and PTM treatments. Bubble size indicates significance (−log(*p*-value)) and color represents activation (red, z-score > 2) or inhibition (blue, z-score < −2). Pathways with *p* < 0.05 are shown.

In the pathway analysis (Figure 14a), both DE and PTM treatments revealed common pathways significantly influenced by Ar-CAP-generated reactive oxygen and nitrogen species (RONS). NGF-stimulated transcription, the most prominently upregulated pathway, indicated that RONS may enhance nerve growth factor (NGF)-mediated signaling, potentially contributing to cellular stress responses and growth regulation. Following NGF, NOD1/2 signaling emerged as another highly significant pathway, suggesting that RONS-induced oxidative stress might activate this pathway as part of a cellular stress response. While NOD1/2 typically participates in immune responses, oxidative stress from Ar-CAP could also trigger this pathway in cancer cells, helping to manage oxidative damage and maintain survival under stress. Additional DE and PTM overlapping pathways, including HMGB1 signaling, the regulation of the epithelial–mesenchymal transition (EMT) by growth factors, and IL-4 and IL-13 signaling, indicated the broader impact of RONS on the tumor microenvironment. These pathways suggest that oxidative stress and inflammation triggered by RONS not only influence cancer cell proliferation and survival but may also modulate inflammatory signaling that alters cell behavior and possibly elicits immunogenic cancer cell death (ICD) in response to the Ar-CAP treatment.

DE-specific pathways involved in Colorectal Cancer Metastasis Signaling and CXCR4 signaling suggested that short-lived RONS generated during DE treatment might trigger cellular stress responses and activate migration-related signaling pathways.

In the functional analysis (Figure 14b), both DE and PTM treatments showed upregulation in pathways related to transcription, cell differentiation, and angiogenesis, suggesting that RONS generated by Ar-CAP might have broadly enhanced gene expression and structural adaptation within the cells. The transcription of RNA and activation of endogenous DNA promoters highlighted increased cellular activity, likely reflecting cellular efforts to manage or respond to oxidative stress. In addition, the upregulation of cancer stem cell proliferation and other tumor-related functions might indicate how the cancer cells were influenced under RONS stress, hinting at selective pressures that could impact survival strategies.

DE-specific functions showed distinct upregulation in pathways related to endothelial tissue development, cell-cell contact, cytoskeleton organization and EMT. These functions suggested that RONS in DE treatment might specifically influence structural adaptation and mobility within cells. The upregulation of processes like cell movement, microtubule dynamics, and EMT in colorectal cancer cell lines further supported this, indicating increased cellular reorganization and migration. Notably, while both DE and PTM treatments contributed to migration phenotypes, only DE demonstrated this effect in NGS results.

In summary, comparing DE and PTM treatments revealed that both target key pathways affected cancer cell behavior through RONS. However, DE had a more immediate and pronounced effect, influencing functions such as cell migration and cytoskeletal organization. In comparison, PTM might induce more gradual and sustained changes. This distinction highlights the importance of further investigating the roles of short- and long-lived RONS in Ar-CAP-based cancer therapies, as their differing durations and functional outcomes could significantly impact therapeutic effectiveness.

### 3.10. Enhanced Effects of Sequential Treatments

Dayun Yan et al. found that, although direct exposure to plasma for 1 or 2 min without further incubation did not affect the viability of PA-TU-8988T cells, the lethal effects of PTM could be enhanced when cells were directly exposed under plasma in advance [41]. The mechanism was not clear.

From the functional analysis of gene expression in this study, it was found that DE treatment induced more gene expression involved in cell behaviors in the early stage than did PTM. We reasonably speculated that short-lived RONS triggered cells to regulate their behaviors to respond to survival stress. This stress in the early stage might be partially overcome over time due to the confined existence of short-lived RONS and their reaction mode to cells. However, it made cells intolerant to the subsequent treatments.

**Figure 15:**
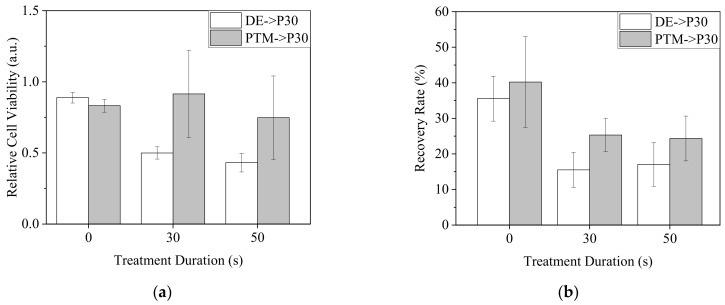
A2058 cells were treated by DE0, 30 and 50 or P0, 30 and 50 with 100 μL of medium for 20 min. The medium was replaced with P30 and the cells were further incubated for 20 h for viability assay (**a**) and 18 h for mobility assay (**b**).

To test the hypothesis, we treated A2058 cells with DE30, DE50, P30 and P50 for 20 min and replaced the medium with P30 with a medium value of 100 μL. MTT and scratch assay were performed to determine the cell viability and mobility, respectively (Figure 15). While the pre-treatment of PTM did not obviously influence the effect of P30 on cell viability, the pre-treatments of DE resulted in significantly lower cell viability (Figure 15a). On the other hand, although the pre-treatment of PTM enhanced the inhibition of cell mobility by P30, DE-pre-treated cells showed even lower mobility than that of PTM-pre-treated cells (Figure 15b). In individual treatments, DE and PTM showed similar influences on cell mobility.

## 4. Discussion

In this study, we measured the excited species in a free jet by OES, the accumulate •OH concentrations by TA and the concentrations of long-lived RONS including H_2_O_2_, NO_2_^−^ and NO_3_^−^ to understand the impact of Ar-CAP generated RONS on cancer cells and fibroblasts in medium and PBS.

Several possible reasons may explain why the fluorescence of HTA could not be significantly measured in the determination of •OH content in DMEM/10% FBS medium: (1) it could be caused by quick depletion of •OH from the interactions between •OH and the complex components in medium, (2) the fluorescent emission of HTA was photoabsorbed by the components in the medium, or (3) the solubility of TA in the medium was too low to synthesize detectable HTA. Since the standard sample of the medium containing 2 μM HTA revealed detectable fluorescence, the first and third assumptions are more likely. However, when the medium was treated by plasma, HTA fluorescence was still undetectable in the TA-containing agarose gel underneath. Here, TA was dissolved in agarose solution but not in the medium, so the solubility of TA in the medium could be ignored. In comparison with the treatments with PBS, HTA fluorescence in agarose gel could be detected at all volumes of 30, 50, 100 and 150 μL; in fact, the obviously increased fluorescence intensity detected in the treatment of the medium indicated that the depth of the liquid was irrelevant in these experiments. Therefore, we speculated that •OH would be consumed quickly in culture medium. Sameer Kalghatgi et al. operated dielectric barrier discharge (DBD) plasma to directly or indirectly treat MCF10A cells through PBS, bovine serum albumin (BSA)-containing PBS and culture medium. The results showed that the indirectly treated PBS did not cause DNA damage. They also treated the cells indirectly with solutions containing different amino acids and found that the degree of DNA damage was correlated to the peroxidation efficiency of the amino acids [39]. The peroxidation of amino acids and proteins might explain the quick depletion of •OH, a highly reactive radical. Besides •OH, other RONS might also interact with the organic molecules in solutions and cells and further affect cell behaviors.

The concentration of H_2_O_2_ increased sharply with treatment times. H_2_O_2_ can form from the dissociation of water molecules and the reactive species and electrons in plasma [23]. The high intensity of •OH in our Ar-CAP might have been the source of the high formation of H_2_O_2_ since the dimerization of •OH is considered to be the major source of H_2_O_2_. By comparison, the intensity of reactive nitrogen species was much lower and therefore led to much lower concentrations of NO_2_^−^ and the rare NO_3_^−^.

When A2058 cells were treated by plasma with different volumes of medium, we found that when 50 and 100 μL of medium were used, direct and indirect treatments caused different degrees of cell death, but when 30 and 150 μL of medium were used, although the lethal effects of direct and indirect treatments increased along with the increases in treatment durations, they exhibited similarity. Considering the depletion of short-lived RONS in medium, their penetration depth might be shallow. The depth of the largest volume, 150 μL, in this work could have been too deep for short-lived RONS to contact cells in the medium. The results of direct and indirect treatment were therefore similar. Since the physical factors to which the cells were exposed were different in these two modalities, it appears that in our system, physical factors had minor or no effects on cell viability.

Interestingly, with the smallest volume of 30 μL and the shallowest depth, cells could be expected to have the greatest chance of contacting short-lived RONS, but the results of direct and indirect treatments were not significantly different. The emission of •OH (λ = 309) on OES and the images of Ar-CAP revealed that the closer the liquid surface was to the plasma outlet, the greater the plasma and •OH emission intensities were enhanced. One of the major generations of •OH by plasma is from the dissociation of water from ambient air by interacting with electrons. At the plasma–liquid interface, the gas flow quickly disturbs the liquid surface, promoting the interaction between plasma and liquid and vaporized water, accelerating the generation of OH radicals. We speculated that when 30 μL of medium was used, the liquid surface was farther from the outlet than it was from the other volumes, and the plasma was thus weaker. Most of the short-lived RONS interacted with the molecules in the medium and could not reach and affect the cells directly, thus leading to similar results between direct and indirect processing.

The change in plasma intensity caused by the distances between the liquid surface and plasma outlet could also explain the stronger lethal effects when 150 μL of medium was used. On the other hand, an appropriate medium depth combined with the plasma intensity might have resulted in excessive short-lived RONS which did not interact with organic molecules but affected cell viability directly in 50 and 100 μL of medium.

Compared with using culture medium, using PBS as the solution in direct plasma treatment seemed to have a better chance of allowing short-lived RONS including •OH to contact the cells. We used 30 μL of PBS for direct and indirect plasma treatments on A2058 cells, and the results showed that the DE treatments caused lethal effects of much greater severity than the indirect treatments. This difference indicated A2058 cells might be intolerant to short-lived RONS. Surprisingly, the cell viability of DE treatments did not differ significantly in relation to the treatment durations. We assumed that this consistency was due to the change in PBS volume by flow-caused evaporation and the tolerance range of A2058 cells to RONS. However, in our previous study, when A2058 cells were treated by DE in PBS for 1 min and the PBS was replaced with fresh medium immediately after the treatment, cell viability decreased, but not as significantly as in the current study [36]. We assumed that a major portion of the short-lived RONS oxidized and damaged the cell membrane, including the membrane proteins and lipids. The damage to the membrane further promoted the effects of long-lived RONS. Since the distributions and patterns of membrane proteins and the composition of lipids differ from cell to cell, the tolerances of cells to short-lived RONS could vary. On the other hand, under certain conditions, the damage to the cell membrane might not be sufficiently severe for cells to undergo apoptosis or necrosis, so the cell viability was not affected significantly when long-lived RONS were removed rapidly.

In contrast to A2058 cells, A549 cells directly and indirectly treated by Ar-CAP with 100 μL of medium were tolerant to the treatments and had high survival rates. However, when the treatments were performed with PBS, cell viability decreased, and DE treatments showed stronger effects. The possible reasons for this could be that the starvation in PBS made A549 cells more sensitive to plasma treatments or that the A549 cells were more susceptible to the species generated by the reactions between PBS and plasma treatments.

With the treatments using 30 μL of PBS, direct treatment possessed stronger lethal effects on BCC cells but not on U2OS cells, NIH3T3, or L929 fibroblasts. NIH3T3 cells were tolerant to the treatments, but the viability of L9292 cells was slightly reduced with longer treatment durations. These results implied that the different cancer cells had different tolerances to short-lived RONS as well as selectivity to the plasma treatments. RONS participate in many signal transduction pathways on the physiological level. The middle accumulation of RONS triggers transformation of cells and high level of RONS drives programmed cell death such as apoptosis, ferroptosis and autophage [42]. The selectivity of RONS-based therapies may be attributed to the differences between cancer and normal cells, including cancer cells bear higher level of RONS and express more aquaporins to deliver RONS [43]. The integration and composition of cell membrane may result in more pore formation or higher fluidity and permeability in cancer cells under plasma treatments [44].

Overall, direct and indirect treatments affected cell viability differently in certain conditions. Under the same conditions, different cells might have different responses. Simple components in treatment solutions such as PBS might reduce the consumption of short-lived RONS.

The NGS analysis revealed significant changes in gene expression following DE and PTM treatments, though it was unclear whether the 2.5 h post-treatment period captured stable cellular responses or reflected ongoing stress. Given the rapid generation of RONS, the observed gene profile might represent an acute stress response rather than long-term adaptation. This timing was crucial for interpreting the results, as short-lived RONS in DE-treated cells might trigger signaling pathways still evolving at the time of sampling. The upregulation of stress-related pathways suggested oxidative damage, but whether these responses stabilized or continued to fluctuate remains unclear. Future studies with longer observation times could help distinguish between transient stress and stable adaptations.

Furthermore, the differences in gene expression between DE and PTM treatments highlight the distinct roles of short- and long-lived RONS in influencing cellular behavior. While DE treatment induces more immediate changes, PTM treatment may allow cells to recover or adapt more gradually, as reflected by the lower number of differentially expressed genes. This suggests that the type of RONS exposure plays a key role in shaping cellular responses.

Based on our assumption that a non-negligible portion of the short-lived RONS reacted with cell membranes, in addition to the genetic analysis showing that DE induced more gene expression for regulating cell behaviors, subsequent treatments would be able to boost the plasma effect on cancer inhibition. When the cells were not continuously cultured in the treated medium after plasma treatments, but the medium was replaced with P30, DE-treated cells were more sensitive to the lethal effect of P30. This result is consistent with a previous study [41]. This could also be owed to the oxidative stress caused by cells intaking both short-lived and long-lived RONS simultaneously from DE treatment, as when intaking only long-lived RONS from PTM treatment, the pre-treatment with DE and PTM would cause different basal levels of RONS in cells and consequently make DE-treated cells more susceptible to overdosing on RONS in subsequent treatments in fresh PTM. The different effects of pre-treatments of DE and PTM on A2058 cell mobility might have been caused by the same factor. However, when the measurement of RONS revealed proportionality to treatment durations, the pre-treatments of 30 and 50 s did not show obvious differences. The dosage might not explain all the phenomena. The preferential targets of the interactions between RONS and cellular molecules might determine the cellular responses. Based on the results, pre-treatment or sequential treatments could be a privileged modality to promote the anti-cancer effects of plasma, and pre-treatment with DE could enhance the effect more effectively than PTM.

In summary, the detected increase and undetectable concentration of HTA in PBS and DMEM/10% FBS, respectively, implied the lethality difference in the DE treatments with various interfaces. We postulate that, due to the limited penetration depths, the exogenous OH radical and other short-lived RONS contact and react with cell membrane before penetrating into the cells. If the treated solution is removed at this stage, when the RONS have not damaged the intracellular molecules, cells may have an activated repair mechanism such as endocytosis and exocytosis to mend the injured membrane and restore homeostasis. However, the sequential treatments make it possible to allow more short-lived and long-lived RONS to enter cells before cells complete the repair and thus achieve better therapeutic effects.

Given the complexity of the interactions among plasma, solutions and cells, it is very difficult to clarify the mechanism governing how plasma inhibits cancer cells. When we just focus on the effects of RONS, there are several questions: (1) Do short-lived and long-lived RONS affect cells in different ways? (2) Is the cancer cell deactivation caused by their combined or synergetic effects? (3) Are there major short-lived or long-lived RONS which play key roles in cancer cell lethality? (4) If RONS share equal effects, is the total oxidative stress more important in cancer inhibition? Although many studies have been carried out to answer these questions, different cells reveal various tolerances under plasma treatments. More evidence from diverse perspectives is required to explain the effects and to pave the way for clinical application of plasma medicine in cancer therapy.

## 5. Conclusions

This study aimed to investigate the effects of Ar-CAP on cell viability and to understand how to increase the anti-cancer efficacy of plasma-generated short-lived RONS. According to OES, Ar-CAP produced high levels of •OH. Meanwhile, the optical emission intensity of Ar-CAP increased when liquid was used as the subject in comparison to a free jet, and as the distance between the plasma outlet and the liquid surface decreased, the intensity gained in strength. HTA from the reaction between •OH and TA could only be detected in PBS or the agarose gel underneath, but not in the medium or the gel underneath. Furthermore, the long-lived RONS, including NO_2_^−^ and NO_3_^−^, were generated in both PBS and medium.

The direct and indirect Ar-CAP treatments of A2058 cells exhibited different effects with 50 and 100 μL of medium, but they exhibited similar effects on viability with 30 and 150 μL of medium. DEB and PTB with 30 μL of PBS showed different effects on A2058, A549 and BCC cells but not on U2OS cells, NIH3T3 or L929 fibroblasts. These results implied the effects of short-lived RONS and the different tolerances of various cells to plasma treatments. Compared to the effects on cancer cells, these treatments resulted in minor or no lethal effects on fibroblasts.

DE and PTM treatments might result in different gene expression profiles due to the distinct impacts of short- and long-lived RONS. In this study, DE treatment exerted more immediate effects, leading to significant cellular stress and damage, ultimately inhibiting cancer cell growth. In contrast, PTM treatment induced more gradual changes, which could influence cancer cell behavior over time. These findings underscore the potential for tailored Ar-CAP therapy based on the differential effects of RONS in cancer treatment.

A clinically executable strategy that possibly allows short-lived RONS to exert distinct cancer suppressive effects by sequential treatments is interpreted. The pre-treatments of DE showed advantages over PTM in cancer inhibition.

This study adds a piece to the overall puzzle, filling only a small gap. More evidence is required to elucidate completely the whole mechanism of plasma treatment on cancer therapy and to determine whether a broad modality for cancer treatment is a possibility or specific treatments for individual cancers are needed for clinical application of plasma medicine.

## Data Availability

The original contributions presented in this study are included in the article. Further inquiries can be directed to the corresponding author.
